# SUMOylation Evoked by Oxidative Stress Reduced Lens Epithelial Cell Antioxidant Functions by Increasing the Stability and Transcription of TP53INP1 in Age-Related Cataracts

**DOI:** 10.1155/2019/7898069

**Published:** 2019-06-10

**Authors:** Bo Lu, Ian T. Christensen, Tao Yu, Chunxia Wang, Qichang Yan, Xinling Wang

**Affiliations:** ^1^Department of Ophthalmology, The Fourth Affiliated Hospital of China Medical University, Key Laboratory of Lens Research of Liaoning Province, Eye Hospital of China Medical University, Shenyang City, Liaoning Province 110005, China; ^2^University of Utah School of Medicine, 30 N. 1900 E, Salt Lake City, Utah 84132, USA; ^3^Department of Medical Imaging, Cancer Hospital of China Medical University, Liaoning Cancer Hospital & Institute, Shenyang City, Liaoning Province 110042, China

## Abstract

Oxidative stress plays an important role in the pathogenesis of cataracts. Small ubiquitin-like modifier (SUMO) proteins have great effects on cell stress response. Previous studies have shown that TP53INP1 can arrest cell growth and induce apoptosis by modulating p53 transcriptional activity and that both TP53INP1 and p53 are substrates of SUMOylation. However, no previous research has studied the effect of SUMOylation on the oxidative stress response in cataracts. This is the first study to investigate the effect of SUMOylation of TP53INP1 in oxidative stress-induced lens epithelial cell injury and age-related cataract formation. We found that the oxidative stress-induced endogenous SUMOylation of TP53INP1 promoted human lens epithelial cell (holed) apoptosis and regulated hLEC antioxidant effects by increasing the stability and transcription of TP53INP1 in age-related cataracts. SUMO-1, SUMOylation, and TP53INP1 were upregulated in lens tissues affected by age-related cataracts. A SUMO-1-specific protease, SENP1, acted as an oxidative stress-sensitive target gene in hLECs. This study identified for the first time that TP53INP1 can be SUMOylated in vivo, that the SUMOylation of TP53INP1 is induced by oxidative stress, and that SUMOylation/deSUMOylation can affect the stability and transcription of TP53INP1 in hLECs.

## 1. Introduction

Cataracts remain the leading cause of blindness worldwide [1]. Unfortunately, the pathogenesis of cataract is still unclear. One universally recognized aspect of noncongenital cataract pathogenesis is that it is always preceded by lens epithelial cell apoptosis [2, 3]. Previous research has clearly demonstrated that various stimulating factors induce the production of reactive oxygen species in the lens and that these species are an important factor in the development of cataract [4, 5]. It has also been demonstrated that cataract patients have significantly increased levels of reactive oxygen species in the anterior chamber and lens [6]. In vitro studies have further demonstrated that hydrogen peroxide equal to that in the lens of cataract patients caused lens epithelial cell apoptosis and lens opacity, changes similar to the pathological process in cataract patients [7, 8].

Small ubiquitin-like modifier-1 (SUMO-1) is an 11 kDa protein with homology to ubiquitin that can covalently bind to target proteins. Posttranslational modification by SUMOs is a reversible process that appears to be involved in the functional regulation of target proteins, including transcriptional regulation, responses to extracellular stimuli, protection against degradation, protein-protein interactions, and subcellular localization [9–15]. SUMOs also play an important role in the cell stress response, and many cell stresses lead to an increase in the formation of SUMO conjugates [16–18].

Tumor protein 53-induced nuclear protein 1 (TP53INP1) is both a p53 cofactor and a p53 target gene, and consequently, its expression is increased in response to several physical and chemical stresses [19]. TP53INP1 contributes to the stress response by regulating the transcriptional activity of p53 and mediating the antioxidant activity of p53 [20]. Previous studies have shown that TP53INP1 induces cell growth arrest and apoptosis by modulating p53 transcriptional activity [21–23] and that both TP53INP1 and p53 are substrates of SUMOylation [24, 25].

Despite previous evidence linking SUMOylation/deSUMOylation with TP53INP1 and the oxidative stress response, there are scant studies focused on their role in cataract formation. In the present study, anterior lens capsules of age-related cataract patients were used to assess the potential effects of SUMOylation/deSUMOylation of TP53INP1 in cataract formation. We also further investigated the molecular mechanisms of SUMOylation/deSUMOylation regulating hLEC apoptosis and the oxidative stress response.

## 2. Materials and Methods

### 2.1. Specimens

We obtained 87 fresh anterior lens capsules from age-related cataract patients with no other eye diseases during phacoemulsification at the Fourth Affiliated Hospital of China Medical University, as well as 69 normal anterior lens capsules from the Eye Bank of the Fourth Affiliated Hospital of China Medical University. This study was approved by the Ethical Committee of the Fourth Affiliated Hospital of China Medical University. All patients provided written informed consent.

### 2.2. Real-Time Quantitative Polymerase Chain Reaction (RT-qPCR)

Total RNA was extracted from tissues and cells using the TRIzol Reagent (Invitrogen, USA) and reverse transcribed using the PrimerScript RT reagent kit (Takara, China) according to the manufacturer's instructions. RT-qPCR was performed using the TaqMan Universal Master Mix II kit (Applied Biosystems, USA), with *β*-actin designated as an endogenous control. The primer sequences are as follows: SUMO-1 forward: 5 ′-TTGGACAGGATAGCAGTGAGATTC-3 ′, SUMO-1 reverse: 5 ′-TCTTCCTCCATTCCCAGTTCTT-3 ′; p53 forward: 5 ′-CAGCAGTCAAGCACTGCCAAG-3 ′, p53 reverse: 5 ′-AGACAGGCATGGCACGGATAA-3 ′; TP53INP1 forward: 5 ′-GCACCCTTCAGTCTTTTCCTGTT-3 ′, TP53INP1 reverse:5 ′-GAGAAAGCAGGAATCACTTGTATC-3 ′; CBX4 forward: 5 ′-CGTCGTTCCAATGTCCTGAC-3 ′, CBX4 reverse: 5 ′-GTAGTACTTGCCGCTCTTGC-3 ′; PIAS3 forward: 5 ′-GGTTTGAGGAAGCGCACTTTA-3 ′, PIAS3 reverse: 5 ′-CTCCTGGCAGAACCTCTCT-3 ′; SENP1 forward: 5 ′-TTCCTCGCTGATGACAACTG-3 ′, SENP1 reverse: 5 ′-AGTGAGTCCATAAGTAGGATACAAGGT-3 ′; and *β*-actin forward: 5 ′-CATCCGTAAAGACCTCTATGCCAAC-3 ′, *β*-actin reverse: 5 ′-ATGGAGCCACCGATCCACA-3 ′. PCR was performed on an ABI 7500 Real-Time PCR System (Applied Biosystems, USA). Three independent experiments were performed, and 2^*ΔΔ*Ct^ quantitative analysis was performed to analyze relative expression levels.

### 2.3. Immunohistochemistry

The fresh anterior lens capsules were immediately fixed with 4% paraformaldehyde in PBS for 30 min at room temperature, permeabilized with 0.5% Triton X-100 for 20 min at room temperature, and treated with 3% hydrogen peroxide/deionized water buffer to inhibit endogenous peroxidase. Then, the fixed capsules were blocked with 10% normal goat serum in PBS and then incubated with anti-SUMO-1 (ab32058; Abcam, USA) antibody in PBS supplemented with 10% normal goat serum overnight at 4°C. Secondary antibody conjugated to horseradish peroxidase (Cell Signaling Technology, USA) was then applied for 1 h at room temperature. Immunoreactivity was detected using diaminobenzidine (DAB; Cell Signaling Technology, USA) and then counterstained with hematoxylin and coverslipped with permount. Immunostaining images were captured using a fluorescence microscope (TH4-200; Olympus, Japan).

### 2.4. Cell Culture and Transfection

Our human lens epithelial cell line (SRA01/04) was generously donated for experimental use by Dr. Yi-sin Liu of the Doheny Eye Institute. SRA01/04 cells were cultured in Dulbecco's modified Eagle's medium (DMEM; Gibco, USA) supplemented with 10% fetal bovine serum (FBS; Gibco, USA), 100 U/mL penicillin, and 100 mg/mL streptomycin (Thermo Scientific, USA) and placed in a 37°C, 5% CO_2_ humidified incubator. SRA01/04 cells were seeded in a 24-well cell culture plate for 24 h. When 80-85% confluence was reached, Lipofectamine 3000 Transfection Reagent (Invitrogen, CA) was used according to the manufacturer's instructions to transfect the cells with pEGFP-SUMO-1, pEGFP-CBX4, and pFlag-SENP1 individually and pEGFP-C1 was used as control. The subsequent experiments were performed 72 h after the completion of transfection [26].

### 2.5. Western Blot Analysis

Total protein was extracted using RIPA lysis buffer with protease inhibitor cocktail (Pierce, USA), and a BCA kit (Thermo Scientific, USA) was employed to quantify protein concentration. 40 *μ*g of protein per specimen was added to each well of 4-12% NuPAGE Bis-Tris precast gels (Invitrogen, CA) for electrophoretic separation of proteins. Proteins were then transferred to PVDF membranes. The membranes were blocked with 5% nonfat milk for 1 h at room temperature, then incubated with different primary antibodies: rabbit anti-SUMO-1 (Abcam, USA), rabbit anti-TP53INP1 (Abcam, USA), mouse anti-p53 (Abcam, USA), rabbit anti-CBX4 (Santa Cruz Biotechnology, USA), rabbit anti-PIAS3 (Abcam, USA), rabbit anti-SENP1 (Abcam, USA), rabbit anti-GAPDH (Abcam, USA), SUMO-1 polyclonal antibody (Abcam, USA), or TP53INP1 polyclonal antibody (Abcam, USA) at 4°C overnight. Horseradish peroxidase- (HRP-) conjugated goat anti-rabbit IgG (H+L) secondary antibody (Promega, USA) and HRP-conjugated goat anti-mouse IgG (H+L) secondary antibody (Promega, USA) were then added, and samples were incubated at room temperature for 2 h. The protein bands were visualized using an ECL Western Blotting Substrate kit (Pierce, USA) after which analysis of protein bands was conducted using the ImageJ software. Three independent experiments were performed.

### 2.6. Measurement of Endogenous Reactive Oxygen Species (ROS)

A 2 ′,7 ′-dichloro-fluorescein diacetate (DCFH-DA) probe was used to detect fluorescence derived from endogenous ROS in hLECs. 1 × 10^4^ cells were seeded into each well of a 96-well plate and were cultured for 24 h, until cells were observed adhering to the sides of the well. The cells were then exposed to 400 *μ*M H_2_O_2_ for 1 h whereupon the culture medium was aspirated, and 10 *μ*M fluorescent probe DCFH-DA was added to each well. This mixture was then incubated in a 37°C incubator for 20 min. The cells were then washed three times with phosphate-buffered saline (PBS), and their DCF fluorescence intensity value (i.e., the mean fluorescence intensity of DCF, representing the level of intracellular ROS) was read using a multifunctional microplate reader. The excitation wavelength used was 485 nm, and the emission wavelength was set at 530 nm. Three independent experiments were performed.

### 2.7. Cell Viability Assay

Cell viability and proliferation were determined using the CellTiter 96 AQ_ueous_ One Solution Cell Proliferation assay kit (Promega, USA). The reagent contains a tetrazolium compound (3-(4,5-dimethylthiazol-2-yl)-5-(3-carboxymethoxyphenyl)-2-(4-sulfophenyl)-2H-tetrazolium, inner salt; MTS). After treatments, according to the manufacturer's protocols, 20 *μ*L MTS solution was added to each well of the 96-well assay plate containing the cells in 100 *μ*L of the culture medium and the cells were then incubated for 4 h at 37°C, 5% CO_2_. The absorbance of each group was read using an absorbance plate reader set to a 490 nm wavelength. The cell viability rates were calculated according to the following formula: the cell viability ratio (%) = [(As − Ab)/(Ac − Ab)] × 100%, where As is the optical density value at 490 nm (OD490) of the treatment group, Ab is the OD490 of the blank group, and Ac is the OD490 of the control group. Three independent experiments were performed.

### 2.8. Caspase-3 Activity Assay

Caspase-3 activity was detected using a caspase-3 assay kit (Abcam, USA). 48 h after transfection, SRA01/04 cells were exposed to 200 *μ*M H_2_O_2_ for 1 h. Then, in accordance with the manufacturer's instructions, these cells were lysed in 50 *μ*L of chilled Cell Lysis Buffer, incubated on ice for 10 mins, and centrifuged and the supernatant protein concentration was determined using the BCA method. 50 *μ*L of Cell Lysis Buffer containing 100 *μ*g protein was added to each well in a 96-well plate. Then, 50 *μ*L 2x reaction buffer, 0.5 *μ*L 10 mM DTT, and 5 *μ*L caspase-3 catalytic substrate DEVD-p-NA substrate were added to each well. The samples were incubated at 37°C for 2 h. The OD value was obtained using a microplate reader set at 405 nm wavelength. Three independent experiments were performed. The caspase-3 experimental group activity was standardized using the following calculation: (OD value of experimental group − blank well OD)/(OD value of control group − blank well OD) × 100%.

### 2.9. Immunofluorescence and Quantification of Immunofluorescence Colocalization

SRA01/04 cells were plated in 4-well chamber slides and exposed to 400 *μ*M H_2_O_2_ for 1 h. The cells were then fixed for 10 min with 4% paraformaldehyde in PBS at room temperature, followed by permeabilization and blockage in PBS containing 10% (*v*/*v*) normal goat serum and 0.2% (*v*/*v*) Triton X-100 for 30 min, then incubated overnight at 4°C with the following primary antibodies diluted in 10% (*v*/*v*) normal goat serum and 0.2% (*v*/*v*) Triton X-100 in PBS: rat anti-SUMO-1 (ab188281; Abcam, USA) or rabbit anti-TP53INP1 (ab202026; Abcam, USA). The cells were then washed three times in PBS, and secondary antibodies, diluted in 10% (*v*/*v*) normal goat serum and 0.2% (*v*/*v*) Triton X-100 in PBS, were applied for 1 h at room temperature and washed. Secondary antibodies were Alexa Fluor® 488-conjugated goat anti-rat IgG (Invitrogen, CA) or Alexa Fluor®594-conjugated goat anti-rabbit IgG (Invitrogen, CA), used at 1 : 500. After three washes in PBS, nuclei were counterstained with 4 ′,6-diamidino-2-phenylindole (DAPI) for 5 min. The chamber on each slide was then removed, and slides were mounted and visualized under a fluorescence microscope (TH4-200; Olympus, Japan) with a 40x objective lens.

Colocalization was measured using the ImageJ software, which analyzes the intensity of each fluorescent label. The Pearson correlation coefficient was used as a measure of colocalization with values between −1 and +1, with positive values indicating a positive correlation. Statistical analyses consisted of Student's *t*-tests with a significance set at 0.05.

### 2.10. Statistical Analysis

Each experiment was performed independently at least 3 times with similar results. Measurement data were presented as mean ± standard deviation (SD). Differences between the groups were calculated using unpaired Student's *t*-tests. Statistical significant difference was considered at *P* < 0.05. Statistical analysis was done using SPSS 16.0.

## 3. Results

### 3.1. SUMO-1, SUMOylation, TP53INP1, and p53 Were Upregulated in the Anterior Lens Capsules of Age-Related Cataract Patients

**Figure 1:**
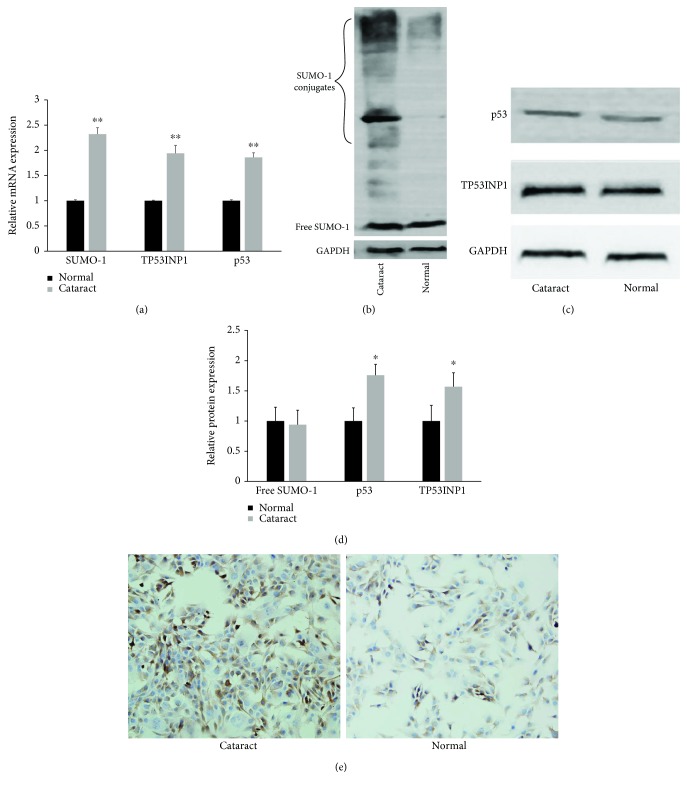
SUMO-1, SUMOylation, TP53INP1, and p53 were upregulated in the anterior lens capsules of age-related cataract patients (cataract) compared with the transparent lens group (normal). (a) The expression levels of SUMO-1, TP53INP1, and p53 mRNA were quantified by RT-qPCR in the two groups. (b) The SUMO-1 conjugates and free SUMO-1 were measured by western blotting in the two groups. (c) TP53INP1 and p53 proteins were measured by western blotting in the two groups. (d) Strip chart of free SUMO-1, p53, and TP53INP1 protein. (e) The immunohistochemistry staining for SUMO-1 in the anterior lens capsules of the two groups. The results are expressed as fold change relative to the normal group. Values represent means ± SE, and *n* = 3. ^∗^*P* < 0.05 and ^∗∗^*P* < 0.001 versus the normal group.

The expression levels of SUMO-1, TP53INP1, and p53 mRNA were quantified by RT-qPCR. Compared with anterior lens capsules of the transparent lens group (normal group), SUMO-1, TP53INP1, and p53 mRNA were significantly higher in the anterior lens capsules of age-related cataract patients (cataract group) (Figure 1(a)). The SUMO-1 conjugates, SUMO-1, TP53INP1, and p53 proteins were then measured by western blotting, and we observed significantly increased SUMO-1 conjugation, TP53INP1, and p53 protein expression in the cataract group while the protein expression of free SUMO-1 did not significantly differ between the two groups (Figures 1(b)–1(d)). Then, the SUMO-1 was stained by immunohistochemistry in the anterior lens capsules of the two groups, and there was increased SUMO-1 in the tissues of the cataract group (Figure 1(e)).

### 3.2. SUMOylation and SUMO-1 Expression Were Enhanced by Oxidative Stress with Increased Expression of ROS and Cell Death in hLECs

**Figure 2:**
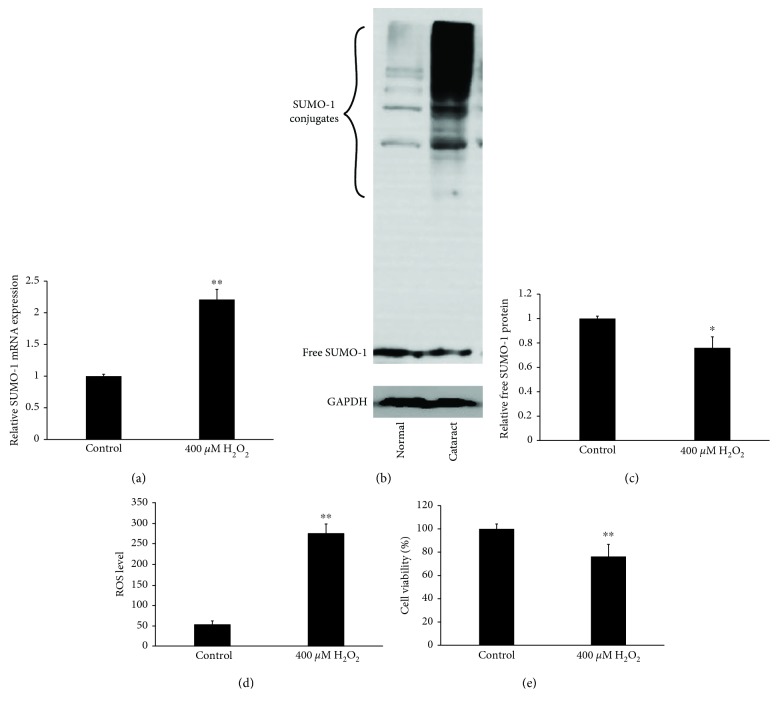
SUMOylation and SUMO-1 expression were enhanced by oxidative stress with increased expression of ROS and cell death in hLECs. (a) The expression level of SUMO-1 mRNA was quantified by RT-qPCR in the two groups and represented as fold change. (b) The SUMO-1 conjugates and free SUMO-1 were measured by western blotting in the two groups. (c) Strip chart of free SUMO-1 protein. (d) The ROS level was quantified by H2DCF dye. (e) Cell viability was quantified by MTS assay. Values represent means ± SE, and *n* = 3. ^∗^*P* < 0.05 and ^∗∗^*P* < 0.001 versus the control group.

Cultured hLECs were exposed to 400 *μ*M H_2_O_2_ for 1 h. Results indicated that compared to the control group, the expression levels of SUMO-1 mRNA detected by RT-qPCR were increased significantly in hLECs treated with 400 *μ*M H_2_O_2_ (Figure 2(a)). Total protein was then extracted and subjected to western blotting analysis with SUMO-1 antibody. A significant increase in SUMO-1 conjugates was observed in cells treated with 400 *μ*M H_2_O_2_, and the increase was correlated with decreased free SUMO-1 (Figures 2(b) and 2(c)), higher expression of ROS quantified by H2DCF dye (Figure 2(d)), and decreased cell viability (Figure 2(e)). These results demonstrate that SUMOylation and SUMO-1 conjugates were enhanced by oxidative stress.

### 3.3. Oxidative Stress Increased SUMO E3 Expression and Suppressed SENP1 Expression

**Figure 3:**
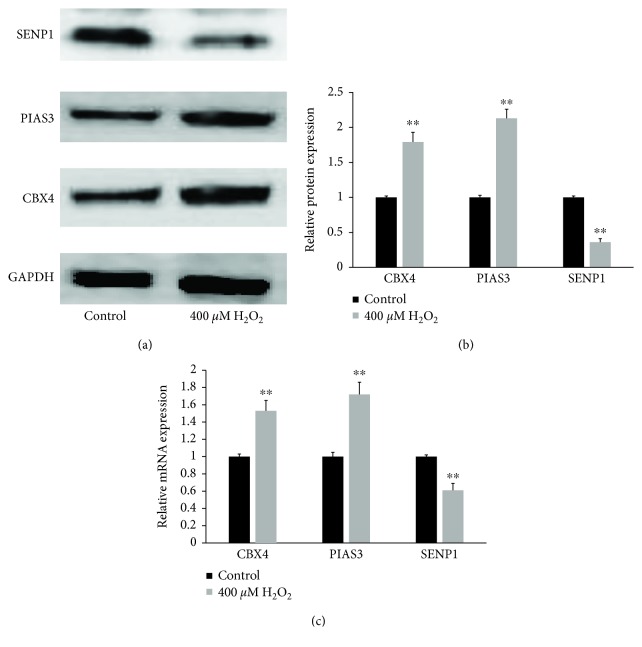
Oxidative stress increased SUMO E3 expression and suppressed SENP1 expression in hLECs. (a) Proteins of CBX4, PIAS3, and SENP1 were determined by western blotting. (b) Strip chart of proteins. (c) mRNA of CBX4, PIAS3, and SENP1 were determined by RT-qPCR and represented as fold change. Values represent means ± SE, and *n* = 3. ^∗∗^*P* < 0.001 versus the control group.

It has been reported that TP53INP1 is SUMOylated by CBX4 and PIAS3 and deSUMOylated by SENP1, 2, and 6 [25]. Based on the results above, we speculated that oxidative stress might also affect SUMO E3 and SENP1 expression. Protein and mRNA isolated from hLECs that were exposed to 400 *μ*M H_2_O_2_ for 1 h and from a control group of hLECs that were not exposed to H_2_O_2_ were processed for western blotting and RT-qPCR. We found that the expression of CBX4 and PIAS3 proteins (Figures 3(a) and 3(b)) and mRNA (Figure 3(c)) was upregulated, whereas the expression of SENP1 protein (Figures 3(a) and 3(b)) and mRNA (Figure 3(c)) was downregulated by oxidative stress.

### 3.4. Oxidative Stress Induced Protein Expression and Transcription of TP53INP1 and Its Transregulator p53 in hLECs

**Figure 4:**
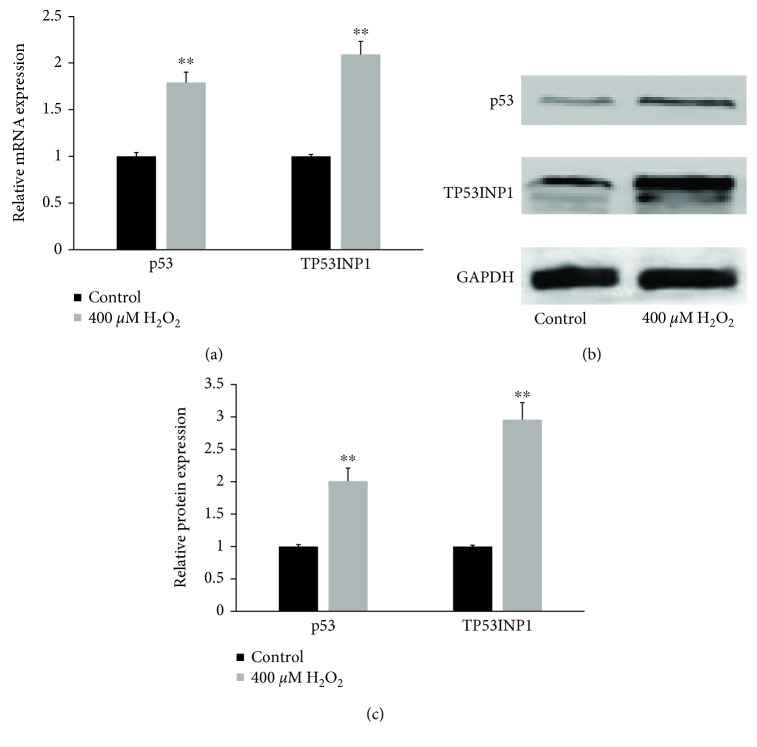
Oxidative stress induced protein expression and transcription of TP53INP1 and p53 in hLECs. (a) mRNA of TP53INP1 and p53 were determined by RT-qPCR and represented as fold change. (b) Proteins of TP53INP1 and p53 were determined by western blotting. (c) Strip chart of proteins. Values represent means ± SE, and *n* = 3. ^∗∗^*P* < 0.001 versus the control group.

To determine whether TP53INP1 is regulated by oxidative stress, hLECs were cultured with 400 *μ*M H_2_O_2_ for 1 h. Then, the expression of TP53INP1 and p53 mRNA was measured using RT-qPCR while the protein expression of TP53INP1 and p53 was observed by western blotting.  Figure 4(a) shows that TP53INP1 and p53 mRNA expression is increased in H_2_O_2_-treated hLECs. The protein expression of TP53INP1 and p53 was also much higher in H_2_O_2_-treated hLECs (Figures 4(b) and 4(c)), and the induction of both was linked to endogenous ROS of hLECs (Figure 2(d)) and cell viability (Figure 2(e)). These results establish that oxidative stress stimulates the expression of the TP53INP1 and p53 mRNA, resulting higher levels of TP53INP1 and p53 protein expression in hLECs.

### 3.5. TP53INP1 Is Targeted by the Endogenous SUMOylation Pathway, Which Is Regulated by Oxidative Stress in hLECs

**Figure 5:**
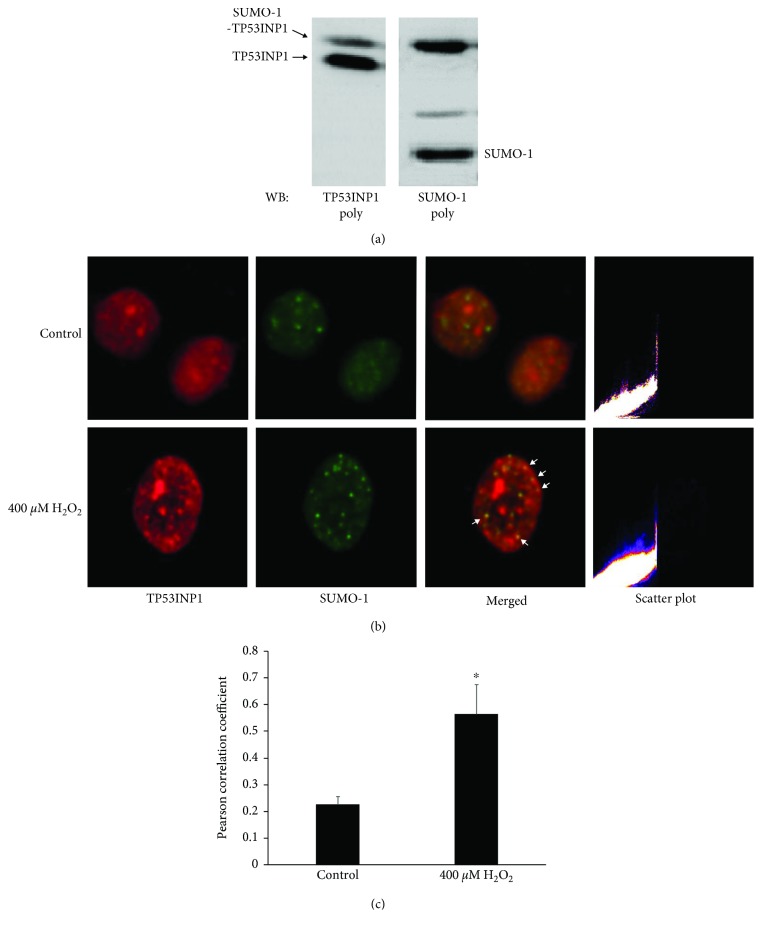
TP53INP1 is targeted by the SUMOylation pathway, which is regulated by oxidative stress in hLECs. (a) SRA01/04 cells treated with 400 *μ*M H_2_O_2_ for 1 h were analyzed by western blot using TP53INP1 polyclonal antibody; then, the membrane was stripped and reprobed with SUMO-1 polyclonal antibody. (b) Immunofluorescence images showing colocalization of TP53INP1 and SUMO-1 in hLECs subjected (400 *μ*M) or not to oxidative stress. (c) Quantification of the colocalization experiments shown in (b). Values represent means ± SE, and *n* = 3. ^∗^*P* < 0.05 versus the control group.

It has recently been discovered that isolated TP53INP1 can be SUMOylated [25]. The aim of this experiment was to determine if intracellular TP53INP1 is modified by endogenous SUMO-1. SRA01/04 cells treated with 400 *μ*M H_2_O_2_ for 1 h were analyzed by western blot using TP53INP1 and SUMO-1 polyclonal antibodies.  Figure 5(a) shows a shifted-up band of approximately ~39 kDa detected by the TP53INP1 polyclonal antibody. Then, to test whether this shift was caused by SUMO-1 conjugation of TP53INP1, we stripped the membrane that had been previously probed with the TP53INP1 polyclonal antibody and reprobed it with a SUMO-1 polyclonal antibody. The shifted-up band was also recognized by the SUMO-1 polyclonal antibody (Figure 5(a)). We therefore surmised that the shifted band represented a SUMO-1-TP53INP1 fusion protein.

Next, we examined whether TP53INP1 and SUMO-1 are colocalized. We used SUMO-1 or TP53INP1 antibody for immunocytochemistry, respectively. Immunofluorescence analysis confirmed the colocalization of the two molecules in the nucleus. Merged images of SUMO-1 (green) and TP53INP1 (red) generating yellow color or granules showed further that a portion of total TP53INP1 protein interacted with TP53INP1 (Figure 5(b)), indicating that only a certain amount of TP53INP1 is SUMOylated. Using this methodology, we evaluated the colocalization of TP53INP1 with SUMO-1 in hLECs that were subjected or not to H_2_O_2_ (Figure 5(b)). The Pearson's_Rr of TP53INP1 and SUMO-1 was 0.23 and increased to 0.57 upon oxidative stress (Figure 5(c)). It indicated that the colocalization of TP53INP1 and SUMO-1 is significantly increased by oxidative stress. In summary, data in Figure 5 indicated that a fraction of TP53INP1 is conjugated to SUMO-1 under physiological conditions and the levels of SUMOylated TP53INP1 are increased following oxidative stress.

### 3.6. Overexpression of SUMO-1 Enhanced the Stability and Transcriptional Activity of TP53INP1 and Its Transregulator p53 in hLECs

**Figure 6:**
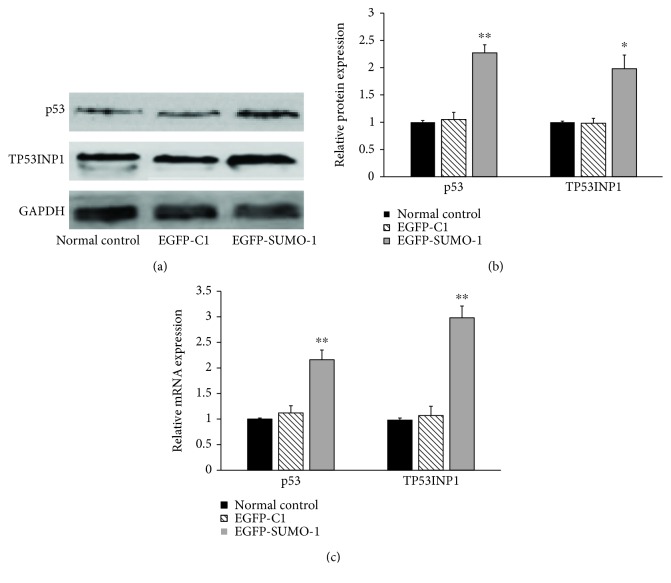
Overexpression of SUMO-1 enhanced the stability and transcriptional activity of TP53INP1 and its transregulator p53 in hLECs. (a) Proteins of TP53INP1 and p53 were determined by western blotting. (b) Strip chart of proteins. (c) mRNA of TP53INP1 and p53 were determined by RT-qPCR and represented as fold change. Values represent means ± SE, and *n* = 3. ^∗^*P* < 0.05 and ^∗∗^*P* < 0.001 versus the normal control group.

The plasmids expressing pEGFP-SUMO-1 were transfected into SRA01/04 cells, and pEGFP-C1 empty vector was transfected as a control. 48 h after transfection, total protein and RNA were extracted from the cells and measured by western blotting and RT-qPCR. As shown in Figures 6(a) and 6(b), the cells overexpressing SUMO-1 displayed increased expression of TP53INP1 and p53 protein. RT-qPCR results showed that SUMO-1 overexpression dramatically upregulated the mRNA of TP53INP1 and p53 (Figure 6(c)), suggesting that SUMO-1 induces the expression of TP53INP1 and p53 at both the protein and mRNA transcription levels.

### 3.7. SUMOylation Upregulated the Stability and Transcriptional Activity of TP53INP1 and p53, Whereas DeSUMOylation Downregulated It in hLECs

**Figure 7:**
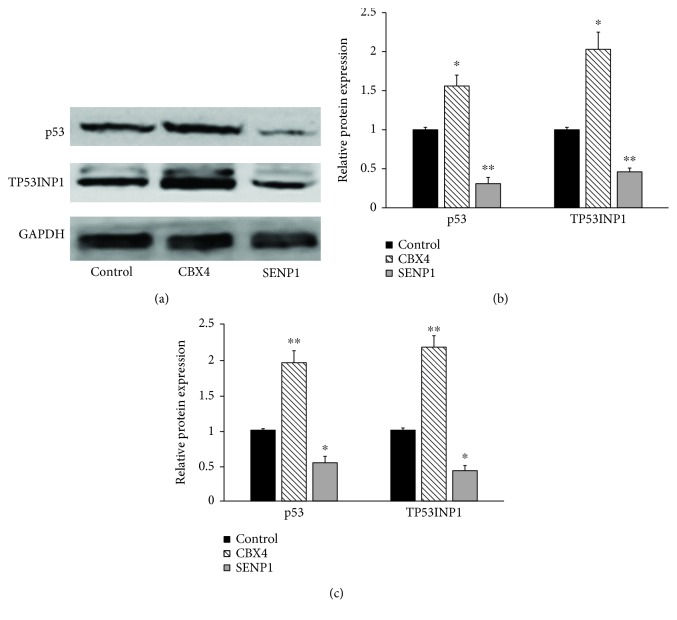
SUMOylation upregulated the stability and transcriptional activity of TP53INP1 and p53, whereas deSUMOylation downregulated it in hLECs. (a) Proteins of TP53INP1 and p53 were determined by western blotting. (b) Strip chart of proteins. (c) mRNA of TP53INP1 and p53 were determined by RT-qPCR and represented as fold change. Values represent means ± SE, and *n* = 3. ^∗^*P* < 0.05 and ^∗∗^*P* < 0.001 versus the control group.

We next examined whether overexpression of CBX4 or SENP1 could affect the expression of TP53INP1 and p53 in hLECs; SRA01/04 cells were transfected with pEGFP-CBX4 and pFlag-SENP1, respectively. Then, expression of TP53INP1 and p53 protein was measured by western blot, and expression of TP53INP1 and p53 mRNA was measured using RT-qPCR. Results showed that cells overexpressing CBX4 displayed a greater abundance of TP53INP1 and p53 protein (Figures 7(a) and 7(b)) and mRNA (Figure 7(c)) than controls. Conversely, cells transfected with SENP1 displayed reduced expression of TP53INP1 and p53 protein (Figures 7(a) and 7(b)) and mRNA (Figure 7(c)). These results indicate that TP53INP1 is highly regulated by the SUMOylation and deSUMOylation machinery, with effects on both its protein expression and mRNA transcription.

### 3.8. SUMOylation/DeSUMOylation Regulated hLEC Proliferation and Apoptosis under Oxidative Stress

**Figure 8:**
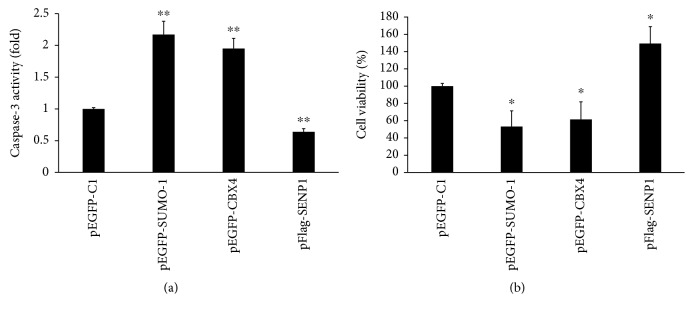
SUMOylation/DeSUMOylation regulated hLEC proliferation and apoptosis under oxidative stress. (a) The caspase-3 activity measured by caspase-3 activity assays and represented as fold change. (b) Cell viability assessed by MTS assays. Values represent means ± SE, and *n* = 3. ^∗^*P* < 0.05 and ^∗∗^*P* < 0.001 versus the pEGFP-C1 group.

SRA01/04 cells were transfected with either pEGFP-SUMO-1, pEGFP-CBX4, pFlag-SENP1, or pEGFP-C1 as a control. 48 h after transfection, the cells were exposed to 200 *μ*M H_2_O_2_ for 1 h, after which caspase-3 activity was assessed and cell viability was determined by MTS assay. Compared with the control group, the SUMO-1 group and the CBX4 group had significantly elevated caspase-3 activity while the caspase-3 activity of the SENP1 group was markedly decreased (Figure 8(a)). These results suggest that SUMOylation promotes apoptosis in hLECs. The MTS assay results showed that compared with the control group, the cell viability in the SUMO-1 group and the CBX4 group was significantly decreased while cell viability significantly increased in the SENP1 group (Figure 8(b)). These results further indicate that SUMOylation inhibits the proliferation of hLECs.

## 4. Discussion

To our knowledge, this is the first study to show the role of SUMOylation and TP53INP1 in oxidative stress-induced lens epithelial cell injury and age-related cataract formation. The major unique finding in the present study is that the oxidative stress-induced SUMOylation and consequent stabilization of TP53INP1 promoted LEC apoptosis and decreased LEC antioxidant activity in tissue affected by age-related cataracts. A further novel finding was that SUMO-1, SUMOylation, and TP53INP1 are upregulated in age-related cataract lens tissues. In this study, for the first time, we directly showed that SENP1, a SUMO-1-specific protease, is an oxidative stress-sensitive target gene in LECs. We also determined for the first time that TP53INP1 can be SUMOylated, that the SUMOylation of TP53INP1 is induced by oxidative stress, and that SUMOylation/deSUMOylation can affect the stability and transcription of TP53INP1 in LECs.

Over the past few years, several studies have indicated that SUMOylation plays an important role in human disease pathogenesis including cancer, neurodegenerative diseases, and heart disease [27–30], but there has been no previous experimental work regarding the role of SUMOylation in age-related cataracts. By comparing healthy anterior lens capsules and those taken from age-related cataract, we found for the first time that the level of SUMO-1 conjugates and the expression of SUMO-1 mRNA were both significantly elevated in the cataract tissues. These findings suggest that SUMOylation may be a key component in the molecular mechanism of age-related cataract formation.

Previous studies have suggested that oxidative damage is a major cause of cataract formation [31] and that p53 protein expression is closely associated with H_2_O_2_-induced oxidative stress [32, 33]. TP53INP1 is a p53 target gene that encodes the TP53INP1 protein, and previous evidence suggests that TP53INP1 may act on p53 via a positive feedback mechanism [20]. Therefore, we postulated that TP53INP1 could be involved in the cataract formation. Our results indicate that the mRNA and protein expression of TP53INP1 and p53 was indeed elevated in age-related cataract lens tissues. This upregulation of TP53INP1 in age-related cataract lens tissues is a novel finding. TP53INP1 is downregulated during tumorigenesis in most cancers, including pancreatic cancer, gastric carcinoma, hepatocellular carcinoma, and melanoma, suggesting that it may play a role in tumor suppression and cell death [34–37]. Previous to our study, there was no previous investigation into the role of TP53INP1 in cataract development. Therefore, this study has important significance in detecting TP53INP1 expression and regulation effects in age-related cataract pathogenesis.

As previously stated, oxidative damage to the lens has been proposed as causes of many types of cataracts, especially age-related cataracts [38]. Cataract patients may have deficient defense systems against factors such as oxidative stress and UV radiation at the onset of the disease [3]. The eye is continuously exposed to environmental stresses including oxidative stress. These stressors induce ROS, which trigger apoptosis in lens epithelial cells, thereby leading to cataract development [39]. However, the exact molecular mechanism of this process is still unclear. Using a human lens epithelial cell line (SRA01/04 cell) as an in vitro model to study the effects of oxidative stress, we found that global SUMOylation was increased and the increase was linked to increased levels of ROS and decreased cell viability. We also determined that elevated expression of SUMO-1 protein and mRNA was also induced by oxidative stress leading us to surmise that SUMO-1 plays an important role in the oxidative stress response in LECs exposed to H_2_O_2_. This supposition is supported by previous reports that oxidative stress led to a dramatic increase of SUMOylation in yeast cells [40]. However, some studies reported that H_2_O_2_ induced deSUMOylation of most substrates at low concentrations in mammalian cells [41]. This apparent contradiction could be explained perhaps by the differential stress conditions in different cell types. Another study consistent with our findings reported that SUMOylation of most proteins increases with aging in the human lens [42]. A previous study conducted in our lab also demonstrated that SUMO-1–4 expression was enhanced by high glucose in LECs [43]. Furthermore, this study further demonstrated that the expression of CBX4 and PIAS3, which are both SUMO E3 ligases, was increased as a result of oxidative stress and that SENP1, a SUMO-1-specific protease, was inhibited by oxidative stress in hLECs. SUMOylation is a reversible process, and target proteins can be SUMOylated by the SUMO E3 ligases and then deSUMOylated by the SUMO-1-specific proteases [25]. Because SUMO itself is a limiting factor for conjugation, SUMOylation is an important process for posttranslational protein modification that influences cell function by regulating the viability of its substrates [44]. Therefore, we postulate that oxidative stress upregulates SUMO-1 expression in order to increase the SUMOylation capacity of LECs. By interacting with its target proteins, this process of SUMOylation then regulates the antioxidant response of the LECs.

p53 is an important regulator not only of cellular apoptosis but also of the cellular antioxidant response [45]. TP53INP1 has also previously been reported to be a major regulator of the p53 response to oxidative stress [20]. In the present study, our results demonstrate that oxidative stress stimulates the expression of TP53INP1 and p53 mRNA, resulting higher levels of TP53INP1 and p53 protein in LECs. We also linked this elevated expression to increased SUMO-1 modification and expression suggesting that SUMO-1 may participate in the antioxidant response of LECs via the SUMOylation of TP53INP1. Cano et al. [20] showed that oxidative stress induces TP53INP1 and p53 in fibroblasts. Kwek et al. [24] reported that p53 is covalently modified by SUMO-1 at lysine 386, while a separate study reported SUMO-1 is conjugated to TP53INP1 on lysine 113 in vitro [25]. Our subsequent studies confirm that intracellular TP53INP1 is modified by endogenous SUMO-1 in vivo and the SUMO modification of TP53INP1 is activated by oxidative stress. Hoege et al. [46] demonstrated that endogenous and exogenous DNA damage induced PCNA ubiquitination and SUMOylation and that these modifications affect the same lysine residue of PCNA and increase resistance to DNA damage. Poliovirus infection increases PML SUMOylation, resulting in the recruitment of p53 and induction of apoptosis [47]. In addition, SUMO can either inhibit or activate transcription during cell stress. Sramko et al. [48] reported that SUMO-2/3 proteins conjugate c-Myb and negatively regulate its stability and activity in cells under stress. Hong et al. [49] reported that stress-induced SUMO-1 modification of HSF1 significantly increases HSF1 activation of HSP gene transcription.

A previous study has shown that TP53INP1 is SUMOylated by the two SUMO ligases CBX4 and PIAS3 and that the three SUMO-1-specific proteases SENP1, SENP2, and SENP6 deconjugate SUMO-1 from TP53INP1 [25]. To further investigate whether SUMOylation/deSUMOylation could regulate the stability and transcriptional activity of endogenous TP53INP1 and p53, SUMO-1, CBX4, and SENP1 were overexpressed, after which the expression of TP53INP1 and p53 protein and mRNA was measured in LECs. Results showed that cells overexpressing SUMO-1 and CBX4 displayed greater abundance of TP53INP1 and p53 protein and mRNA than controls, whereas cells transfected with SENP1 displayed reduced expression of TP53INP1 and p53 protein and mRNA compared with controls. TP53INP1 is a target gene of the transcription factor p53 [50], and TP53INP1 is also able to activate the transcriptional activity of p53 [51]. These results demonstrate that stability and transcriptional activity of TP53INP1 appear to be under the control of SUMOylation and deSUMOylation machinery. Previous research has showed that SUMOs can prevent ubiquitin-mediated degradation of target proteins by competing for lysines, thereby enhancing the stability and transcriptional activity of target substrates [52]. Lee and Kim [14] indicated that SUMO-2 modification is implicated in the modulation of the NDRG1 protein level and function.

In addition to the effect of SUMOylation/deSUMOylation on the oxidative stress response in LECs, in this study, we further report that the overexpression of SUMO-1 and CBX4 significantly upregulated caspase-3 activity and downregulated cell viability, whereas SENP1 overexpression decreased caspase-3 activity and increased cell viability in LECs exposed to oxidative stress. These results suggest that SUMOylation promotes apoptosis, inhibits proliferation, and reduces antioxidant function in LECs and that deSUMOylation can reverse this process. Given the important role that p53 plays in regulating cell apoptosis, cell proliferation, cell differentiation, and DNA repair, as well as its role in the oxidative stress response [53–57], further elucidating its role in cataract development was a central part of our experiment. TP53INP1 is both a p53 cofactor as well as a p53 target gene, and consequently, its expression is similarly induced in response to a variety of physical and chemical stresses [19]. TP53INP1 can act directly on p53, modulating its activity by phosphorylating a serine residue in position 46 (ser-46) [51]. TP53INP1 is also a key transcription factor in the p53 apoptosis pathway, and its expression increases in response to oxidative stress [58]. Okamura et al. [23] found that p53 and related transcription factors are upregulated in cells with stress-induced DNA damage, upregulating the p53-TP53INP1 pathway, which regulates in cell apoptosis. The current study further demonstrates that SUMOylation of TP53INP1 is increased in response to oxidative stress and that this process not only increases TP53INP1 stability but also upregulates its transcription. Therefore, we propose that SUMOylation plays an important role in activating the TP53INP1-p53 pathway, regulating cell apoptosis, proliferation, and the oxidative stress response.

## 5. Conclusion

In conclusion, the findings of the present study demonstrate that oxidative stress increases SUMOylation of TP53INP1 thereby increasing TP53INP stability and transcriptional activity. This then upregulates the TP53INP1-p53 pathway which induces cell apoptosis, inhibits cell proliferation, and reduces antioxidant function in hLECs, thus leading to the development of age-related cataracts.

## Data Availability

The data used to support the findings of this study are available from the corresponding author upon request.
